# Combined Radiographic Features and Age Can Distinguish *Mycoplasma pneumoniae* Pneumonia from Other Bacterial Pneumonias: Analysis Using the 16S rRNA Gene Sequencing Data

**DOI:** 10.3390/jcm11082201

**Published:** 2022-04-14

**Authors:** Yuto Iwanaga, Kei Yamasaki, Kazuki Nemoto, Kentaro Akata, Hiroaki Ikegami, Keigo Uchimura, Shingo Noguchi, Chinatsu Nishida, Toshinori Kawanami, Kazumasa Fukuda, Hiroshi Mukae, Kazuhiro Yatera

**Affiliations:** 1 Department of Respiratory Medicine, University of Occupational and Environmental Health, Japan, Kitakyushu 807-8555, Japan; y-iwanaga@med.uoeh-u.ac.jp (Y.I.); kazuki585@outlook.jp (K.N.); kentarouakata@med.uoeh-u.ac.jp (K.A.); h_i214538y@yahoo.co.jp (H.I.); honorific2006@yahoo.co.jp (K.U.); sn0920@med.uoeh-u.ac.jp (S.N.); c-nishi@med.uoeh-u.ac.jp (C.N.); namihei@med.uoeh-u.ac.jp (T.K.); yatera@med.uoeh-u.ac.jp (K.Y.); 2 Department of Microbiology, University of Occupational and Environmental Health, Japan, Kitakyushu 807-8555, Japan; kfukuda@med.uoeh-u.ac.jp; 3 Department of Respiratory Medicine, Nagasaki University Graduate School of Biomedical Sciences, Nagasaki 852-8523, Japan; hmukae@nagasaki-u.ac.jp

**Keywords:** *Mycoplasma pneumoniae* pneumonia, clone library analysis, 16S rRNA gene, centrilobular nodules, chest computed tomography, bronchoalveolar lavage fluid

## Abstract

The study objective was to evaluate chest radiographic features that distinguish *Mycoplasma pneumoniae* pneumonia (MPP) from other bacterial pneumonias diagnosed based on the bacterial floral analysis with 16S rRNA gene sequencing, using bronchoalveolar lavage fluid samples directly obtained from pneumonia lesions. Patients were grouped according to the dominant bacterial phenotype; among 120 enrolled patients with CAP, chest CT findings were evaluated in 55 patients diagnosed with a mono-bacterial infection (one bacterial phylotype occupies more than 80% of all phylotypes in a sample) by three authorized respiratory physicians. Among this relatively small sample size of 55 patients with CAP, 10 had MPP, and 45 had other bacterial pneumonia and were categorized into four groups according to their predominant bacterial phylotypes. We created a new scoring system to discriminate MPP from other pneumonias, using a combination of significant CT findings that were observed in the *M. pneumoniae* group, and age (<60 years) (MPP–CTA scoring system). When the cutoff value was set to 1, this scoring system had a sensitivity of 80%, a specificity of 93%, a positive predictive value of 73%, and a negative predictive value of 95%. Among the CT findings, centrilobular nodules were characteristic findings in patients with MPP, and a combination of chest CT findings and age might distinguish MPP from other bacterial pneumonias.

## 1. Introduction

*Mycoplasma pneumoniae* pneumonia (MPP) is one of the most common causes of community-acquired pneumonia (CAP) worldwide. Despite a recent increase in the rate of macrolide-resistant *Streptococcus pneumoniae* infection [1], the Japanese Respiratory Society (JRS) guidelines and British Thoracic Society guidelines [2] recommend macrolides as the first line of treatment for MPP. Macrolides, tetracycline, and respiratory quinolones are effective candidates for treating MPP, but cephalosporines and penicillin are ineffective; therefore, it is crucial to distinguish MPP from other bacterial pneumonias because of their different treatment modalities. The JRS guidelines recommend a clinical scoring system that includes age, respiratory symptoms, peripheral white blood cell (WBC) count, and auscultation findings. It distinguishes atypical pneumonia from bacterial pneumonia with relatively high sensitivity (77%) and specificity (93%) [3]. To date, there have been several reports of chest computed tomography (CT) findings of MPP; they have indicated that ground-glass attenuation (GGA) is more frequently observed in patients with MPP than in patients with pneumococcal pneumonia [4]. Furthermore, centrilobular nodules, GGA, and bronchial wall thickening are more frequently observed in patients with MPP than in patients with *S. pneumoniae* pneumonia [5]. 

Pneumonia, including CAP, is usually diagnosed using a sputum smear and/or culture and urinary antigen detection for *S. pneumoniae*, *M. pneumoniae*, and *Legionella pneumophila*. A recent study showed that approximately half of the causative pathogens of CAP are mixed bacteria [6]. Sputum culture, serum antibody titer, and specific urinary antigen detection do not clearly distinguish mono-bacterial from mixed bacterial infections, and there may be mixed bacterial pneumonias that include *M. pneumoniae* pneumonia, which may affect chest CT findings. 

Using the 16S rRNA gene sequencing analysis with a clone library method of bronchoalveolar lavage fluid (BALF) directly obtained from pneumonia lesions in the lung can provide culture-independent, unbiased bacterial floral information [6,7]. Using this analytic method, bacterial species and their proportions in the pneumonia lesion can be detected to determine whether an infection is mono-bacterial (one bacterial phylotype occupies more than 80% of all phylotypes in a sample) or a mixed bacterial infection [6,7]. 

The purpose of the present study was to precisely evaluate the chest CT findings and clinical information of patients with pneumonia who were diagnosed with mono-bacterial pneumonia, confirmed using the 16S rRNA gene sequencing analysis with a clone library method of BALF, and to create a new scoring system to differentiate patients with MPP from those with other bacterial pneumonias. 

## 2. Patients and Methods

### 2.1. Study Participants

Adult patients (aged ≥ 18 years) with CAP, diagnosed at the University of Occupational and Environmental Health, Japan, (UOEH) and 12 related community hospitals from April 2010 to March 2020, were retrospectively assessed. BALF was directly obtained from pulmonary pneumonia lesions in patients with pneumonia. Patients with coronavirus disease 2019 (COVID-19) were not included in this study because enrollment of subjects in this study took place before the start of the COVID-19 pandemic in our region.

### 2.2. Diagnostic Criteria for CAP and Atypical Pneumonia

CAP was defined according to the guidelines of the American Thoracic Society (ATS)/Infection Disease Society of America (IDSA) [8,9]. 

The diagnostic criteria for atypical pneumonia proposed in the JRS guidelines were used, and the criteria were as follows: (i) age less than 60 years; (ii) no or only minor underlying diseases; (iii) persistent cough; (iv) limited chest auscultatory findings; (v) no sputum or no identification of etiological pathogens by rapid diagnostic tools; and (vi) a peripheral WBC count of <10,000/mL. Each parameter was scored as either 0 or 1, and the total score was derived by adding all six scores (ranging from 0 to 6). Patients with scores of 4 or higher were suspected of having atypical pneumonia such as MPP or *Chlamydophila pneumoniae* pneumonia [3].

### 2.3. Sample Collection

A total of 40 mL of BALF samples was obtained directly from pneumonia lesions via fiberoptic bronchoscopy, while minimizing sample contamination by rinsing with povidone 1% iodine solution before bronchoscopy, and avoiding any contact and suction in the oral cavity. The samples were then stored at 4 °C until use, as previously described [6].

### 2.4. Evaluation of Lung Bacterial Flora Using the 16S Ribosomal RNA Gene Analysis

DNA was extracted from each BALF sample by vigorous shaking with glass beads and sodium dodecyl sulfate, and fragments of the V3–V5 regions of the 16S rRNA gene were amplified by polymerase chain reaction (PCR) using universal primers (341F and 907R). After cloning the PCR products using a TOPO TA cloning vector (Invitrogen; Carlsbad, CA, USA) [6,7,10], the sequences of 96 randomly selected colonies from each clone library were determined using a 3130xl Genetic Analyzer (Applied Biosystems, Bedford, MA, USA), with an average read length of 550 bases [6,7,10]. The sequences of the 16S rRNA genes were compared with those of the bacterial type strains, using the Basic Local Alignment Search Tool (BLAST) algorithm to estimate the bacterial phylotype, and a phylotype sharing 97% or higher homology with the type strain sequence was assumed to be a presumptive species, as previously described [6,7,10]. When a certain predominant phylotype comprised over 80% of all detected bacterial phylotypes in a sample, the patient was assigned to the “mono-bacterial” group, and when less than 80% predominance of any one phylotype was observed, patients were assigned to the “mixed-bacterial” group, as reported previously [6,7].

### 2.5. Evaluation of Chest CT Findings

After excluding patients with obvious pulmonary complications that could affect the evaluation of chest CT findings, such as interstitial pneumonia, congestive heart failure, severe pulmonary emphysema, and massive previous tuberculous lesions, chest CT findings (consolidation, GGA, centrilobular nodule, bronchial wall thickening, reticular or linear opacity, and pleural effusion) of patients in the mono-bacterial group were independently evaluated and scored by three authorized respiratory specialists (with 19, 15, and 10 years of clinical experience), blinded to any clinical information about the patients including the clinical severity of the pneumonia, and the final decision for each finding was reached by consensus of all three analysts, as previously described [4]. Chest CT analysis was not performed in the mixed bacterial group to avoid possible interference of multiple bacterial species. Patients with pneumonia were classified according to their dominant bacterial phenotype, and the CT findings of each group were compared; based on these results, we created a new scoring system to differentiate MMP from other bacterial pneumonias. 

### 2.6. Statistical Analysis

The SPSS software package (version 27; IBM Corporation, Armonk, NY, USA) was used for statistical analyses. Continuous variables were compared using the Kruskal–Wallis test, Mann–Whitney U-test and Student’s *t*-test, whereas categorical variables were compared using Fisher’s exact test or the chi-square test, as appropriate. Kappa value was calculated by the respiratory specialists based on radiological findings. We further performed a multivariate analysis using logistic regression with backward elimination. Statistical significance was set at *p* < 0.05.

## 3. Results

**Figure 1:**
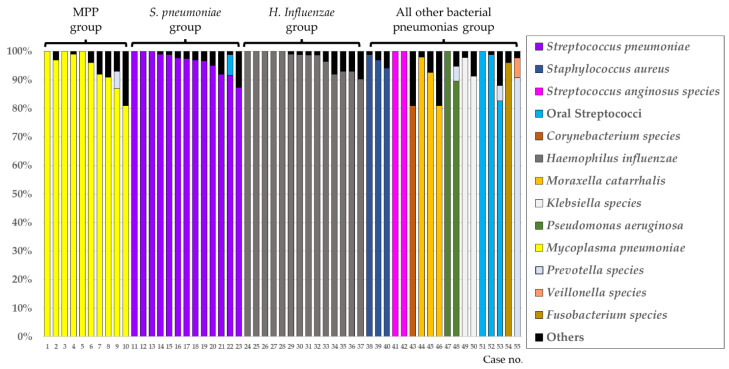
Percentage of detected phylotypes in “mono-bacterial” group using the molecular method. The percentage of phylotypes in each sample in the 55 patients in the mono-bacterial group. The phylotypes that dominated less than 5% in each library were classified as “Others.” Abbreviations: MMP, *Mycoplasma pneumoniae* pneumonia; *S. pneumoniae*, *Streptococcus pneumoniae*; *H. influenzae*, *Haemophilus influenzae*.

Among the 120 enrolled patients with CAP, 57 were assigned to the mono-bacterial group. Two of these 57 patients were excluded because their chest CT findings were unable to be accurately evaluated due to severe heart failure and severe interstitial pneumonia; therefore, 55 patients were eventually evaluated. Among these 55 patients, 10 had MPP, and the remaining 45 had other bacterial pneumonias (*S. pneumoniae* pneumonia, 13; *Haemophilus influenzae* pneumonia, 14; and others, 18) (Figure 1), which were classified into four groups (MPP, *S. pneumoniae* pneumonia, *H. influenzae* pneumonia, and others) according to the predominant bacterial phylotypes.

**Figure 2:**
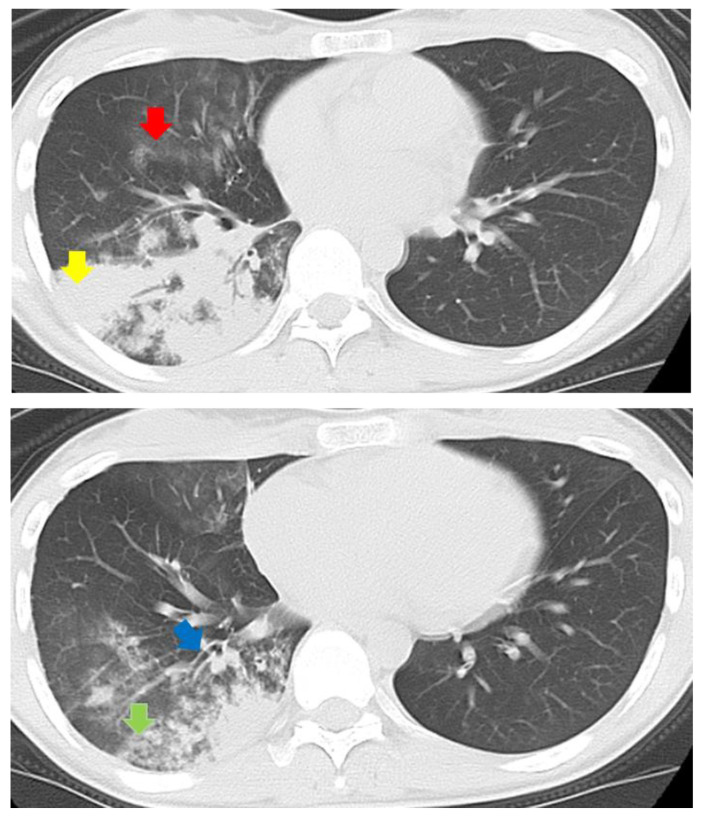
Chest CT of a 24-year-old Japanese woman with MPP showed various findings such as ground-glass attenuation (red arrow), consolidation (yellow arrow), centrilobular nodules (green arrow), and branchial wall thickening (blue arrow).

The baseline characteristics and scoring for atypical pneumonia (Table 1) and chest CT findings (Table 2) of these enrolled 55 patients are shown in Table 1 and Table 2. Among the four groups, age was significantly lower in the MPP group (MPP, 30.6 ± 17.0 years; *S. pneumoniae* pneumonia, 72.9 ± 8.5 years; *H. influenzae* pneumonia, 73.9 ± 11.7 years; others, 65.7 ± 20.5 years; *p <* 0.001). WBC counts (WBC < 10,000/µL) and “scoring for atypical pneumonia (≥4)”, according to the JRS guidelines, were significantly higher in the MPP group, and the pneumonia severity index was not significantly different among the groups (Table 1). Consolidation, GGA, centrilobular nodules, and branchial wall thickening were frequently observed (>80%) in patients in the MPP group. Centrilobular nodules were observed with significantly higher frequency in the MPP group, whereas the occurrence of consolidation, GGA, bronchial wall thickening, reticular or liner opacity, and lymphadenopathy were not significantly different among the groups (Table 2, Figure 2). The kappa value was 0.77 for the analysis of CT findings, indicative of good inter-rater agreement. Next, we selected important baseline characteristic factors (age and sex) and all chest CT findings as variables for the logistic regression analysis. The following variables were entered into a backward stepwise multivariate logistic regression analysis: age < 60 years; sex, consolidation, GGA, centrilobular nodule, bronchial wall thickening, reticular or linear opacity, and pleural effusion. The independent predictor variables for diagnosis of MPP in the multivariable model are shown in Table 3. 

Using these results, we created a new scoring system with two factors, a combination of significant CT findings in the MPP group (centrilobular nodules) and age (<60 years) (“*Mycoplasma pneumoniae* pneumonia-CT and Age [MPP–CTA] scoring system”), to distinguish MPP from other bacterial pneumonias. 

**Figure 3:**
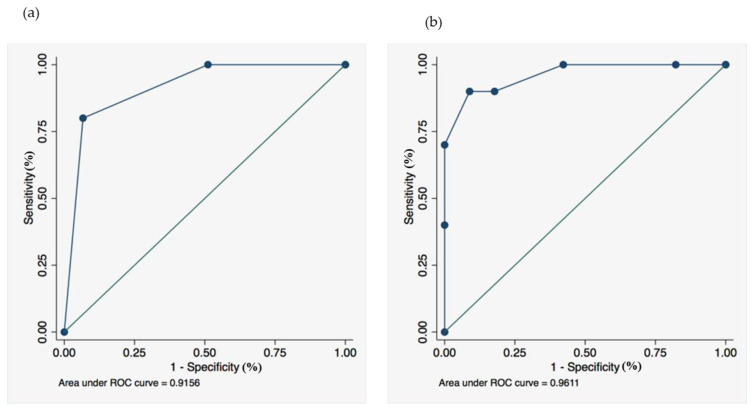
Receiver operating characteristic curves in (**a**) the *Mycoplasma pneumoniae* pneumonia-computed tomography age (MPP–CTA) scoring system and (**b**) the JRS Scoring for atypical pneumonia.

Table 4 displays details of the scores and the average of the total score for the MPP group (*n* = 10) and the other bacterial pneumonia groups (*n* = 45) was 0.90 ± 0.32 and 0.44 ± 0.50, respectively. Receiver operating characteristic (ROC) curves for MPP–CTA and the JRS scoring for atypical pneumonia were constructed. The best diagnostic cutoff (sensitivity = specificity) of each scoring system was 0.79 and 2.88 (Figure 3). When the cutoff value was set to the integer values of one and three for each scoring system, MPP–CTA and the JRS scoring for atypical pneumonia showed a sensitivity of 80% and 90%, a specificity of 93% and 91%, positive predictive values (PPVs) of 73% and 69%, and negative predictive values (NPVs) of 95% and 98%, respectively. The area under the ROC curve was 0.9156 in MPP–CTA (Figure 3a) and 0.9611 in the JRS scoring for atypical pneumonia (Figure 3b). MPP–CTA and the JRS scoring for atypical pneumonia were compared with the DeLong method, and there were no significant differences between the groups (*p* = 0.41).

## 4. Discussion

In the present study, we created a new and simple scoring system (MPP–CTA) that used chest CT findings and age to differentiate MPP from CAPs other than MPP. For this purpose, we used the chest CT data of patients with mono-bacterial pneumonia, diagnosed based on the 16S rRNA gene sequencing analysis, using directly obtained BALF samples from pneumonia lesions. Our method provided precise information about the types of bacteria in the lung lesions in patients with pneumonia, and clinical and radiological information regarding mono-bacterial pneumonia with causative bacterial species. According to our results, centrilobular nodules were significantly more frequently observed in the MPP group than in the non-MPP CAP group, and the new scoring system (MPP–CTA) with a cutoff of one showed a moderate sensitivity (80%), high specificity (93%), and high NPV (95%) in distinguishing MPP from other bacterial pneumonias, similar to the traditional scoring system (JRS scoring for atypical pneumonia), in spite of the relatively small sample size.

A unique aspect of the present study making it valuable for evaluating CT findings of pneumonia caused by certain bacterial species, was that it used culture-independent bacterial floral data of 16S rRNA gene sequencing to precisely identify bacterial pathogens in patients with pneumonia. Several studies have evaluated CT images of patients with pneumonia; however, the diagnosis is generally made from the results of serum tests, sputum culture, or urinary antigen detections. In addition, although pneumonia has been considered to be a single bacterial infection, our observation indicated that about half of the patients with CAP had multi-bacterial infections [6]. Therefore, this is the first study to use mono-bacterial pneumonias diagnosed using highly accurate bacterial floral data of BALF collected directly from pneumonia lesions, and chest CT findings of patients with pneumonia caused by a certain bacterial species. 

With regard to the comparison of radiographic findings of MPP and other bacterial pneumonias, bronchial wall thickening (81% (52/64) vs. 19% (13/68), respectively) and centrilobular nodules (78% (50/64) vs. 22% (32/68), respectively) were significantly more frequently observed in patients with MPP than in patients with *S. pneumoniae* [4]. Another study also showed that centrilobular nodules were significantly more frequently observed in patients with MPP than in patients with *S. pneumoniae* pneumonia (97.6% (41/42) vs. 17.6% (6/34), respectively) [11]. Compared with these previous observations, the presence of centrilobular nodules was significantly more frequent in patients with MPP than in patients assigned to other bacterial groups (90% MPP vs. 54% *S. pneumoniae* vs. 57% *H. influenzae* vs. 28% others) in the present study, and our results are concordant with those of previous studies [4,11]. Centrilobular nodules have been reported to be more significant in MPP than in *S. pneumoniae* pneumonia [4]. However, our study showed that this finding is also a significant characteristic compared to not only *S. pneumoniae* pneumonia but also other bacterial pneumonias such as *H. influenzae* pneumonia.

The diagnostic criteria for atypical pneumonia proposed by the JRS guidelines are broadly used in Japan [3]. In a comparison of our new MPP–CTA scoring system and the JRS criteria for atypical pneumonia, similar sensitivity, specificity, PPVs, and NPVs were obtained when the cutoff MPP–CTA score was 1. Additionally, although the size of this study was relatively small, our system showed slightly higher specificity and PPV than the JRS criteria for atypical pneumonia. Taken together, these findings suggest that our system may help physicians to accurately diagnose pneumonias using chest CT evaluations. 

This study had some limitations. First, the number of patients in this study was relatively small. Studies with a small sample size are sometimes affected by sampling bias, which in this case, might not reflect the real-world proportion of pneumonia patients. Furthermore, this study was only a derivation cohort study to construct an MPP–CTA scoring system and did not include a validation cohort study. A confirmation study should be performed to confirm the utility of this new scoring system. Moreover, the study may have some selection biases because it focused on evaluating the CT findings of mono-bacterial infections identified using BALF obtained from the pneumonia-affected site, and the MPP–CTA scoring system requires chest CT findings, which cannot be applied to patients with a mild pneumonia that does not require a chest CT. Second, the universal primers used in our study were unable to amplify all bacterial 16S rRNA genes, but the sensitivity of the primers was approximately 92% for the registered bacterial species in the Ribosomal Database Project II database. The remaining 8% bacteria did not include human pathogens. Third, this study was retrospective. Fourth, this study did not include patients with viral, pneumocystis, and fungal pneumonias. Further studies to verify the results of the present study are necessary to establish its clinical usefulness in future large-scale prospective studies.

## 5. Conclusions

The newly created MPP–CTA scoring system can sufficiently distinguish MPP from other bacterial pneumonias, and among chest CT findings, the presence of centrilobular nodules are significant characteristic findings in patients with MPP. Although this study included relatively small numbers of patients in each category of bacterial species, this scoring system has sensitivity and specificity similar to those of the Japanese Respiratory Society criteria for atypical pneumonia. A precise diagnosis of MPP may help physicians determine the most appropriate antimicrobial treatment, especially with respect to antimicrobial resistance. Further large-scale prospective validation studies are necessary to establish the clinical usefulness of the MPP–CTA.

## Figures and Tables

**Table 1:** Background characteristics of the 55 patients in this study.

Characteristic	*Mycoplasma pneumoniae*	*Streptococcus pneumoniae*	*Haemophilus influenzae*	All Other Bacterial Pneumonias *	*p*
	(*n* = 10)	(*n* = 13)	(*n* = 14)	(*n* = 18)	
Age (y); mean ± SD	30.6 ± 17.0	72.9 ± 8.5	73.9 ± 11.7	65.7 ± 20.5	<0.001
Sex Female; *n* (%)	6 (60)	5 (38)	9 (64)	3 (17)	0.029
PSI risk					0.059
1–3	9 (90)	8 (62)	5 (36)	9 (50)	
4	0 (0)	2 (15)	8 (57)	6 (33)	
5	1 (10)	3 (23)	1 (7)	3 (17)	
Comorbid diseases					
Malignancy	0	3	1	4	
COPD	0	3	4	7	
Bronchiectasis	0	0	5	4	
Interstitial pneumonia	0	0	2	1	
Cerebrovascular disease	0	0	3	3	
Diabetes mellitus	0	4	3	2	
Congestive heart disease	0	0	4	0	
Chronic kidney disease	0	0	2	1	
Chronic liver disease	0	1	0	0	
RA or Sjogren’s syndrome	0	0	1	0	
WBC (/uL) < 10,000	9 (90)	5 (38)	7 (50)	6 (33)	0.028
The JRS Scoring for atypical pneumonia (≥4)	9 (90)	1 (8)	1 (7)	2 (11)	<0.001

Abbreviations: COPD, chronic obstructive pulmonary disease; WBC, white blood cell; PSI, pneumonia severity index; SD, standard deviation; RA, rheumatoid arthritis. * Pneumonia by *Staphylococcus aureus*, *Streptococcus anginosus*, *Moraxella catarrhalis*, *Klebsiella* species, *Pseudomonas aeruginosa*, *Prevotella* species, *Veillonella* species, *Fusobacterium* species, and *Corynebacterium* species.

**Table 2:** Chest CT findings of the 55 patients.

Chest CT Findings	*Mycoplasma pneumoniae*	*Streptococcus pneumoniae*	*Haemophilus influenzae*	All Other Bacterial Pneumonias *	*p*
	(*n* = 10)	(*n* = 13)	(*n* = 14)	(*n* = 18)	
Consolidation	9 (90)	10 (77)	13 (93)	15 (83)	0.6572
Grand-glass attenuation	8 (80)	5 (38)	7 (50)	10 (56)	0.2515
Centrilobular nodules	9 (90)	7 (54)	8 (57)	5 (28)	0.0171
Branchial wall thickening	10 (100)	9 (69)	10 (71)	11 (61)	0.1670
Reticular or liner opacity	0 (0)	5 (38)	3 (21)	2 (11)	0.0889
Pleural effusion	5 (50)	1 (8)	6 (43)	3 (17)	0.0492
Lymphadenopathy	2 (20)	5 (38)	6 (43)	1 (6)	0.0627

* Pneumonia by *Staphylococcus aureus*, *Streptococcus anginosus*, *Moraxella catarrhalis*, *Klebsiella* species, *Pseudomonas aeruginosa*, *Prevotella* species, *Veillonella* species, *Fusobacterium* species, and *Corynebacterium* species.

**Table 3:** Independent predictor variables for *Mycoplasma pneumoniae* pneumonia of the multivariable model.

Variable		β Coefficient	Odds Ratio	95% Confidence Interval	*p*
Age (years)					
	<60	3.95	52.2	4.97–547.11	0.001
	60 and more than 60		–	–	–
CT findings					
	Centrilobular nodules	2.52	12.5	1.06–146.52	0.0446

Results are presented as odds ratios and 95% confidence intervals.

**Table 4:** *Mycoplasma pneumoniae* pneumonia-computed tomography age (MPP–CTA) scoring system.

	0	1
Age (years)	60 and more than 60	<60
Centrilobular nodules	absent	present

## Data Availability

Not applicable.
